# Defects in Host Immune Function in Tree Frogs with Chronic Chytridiomycosis

**DOI:** 10.1371/journal.pone.0107284

**Published:** 2014-09-11

**Authors:** Sam Young, Paul Whitehorn, Lee Berger, Lee F. Skerratt, Rick Speare, Stephen Garland, Rebecca Webb

**Affiliations:** 1 James Cook University, One Health Research Group, School of Public Health, Tropical Medicine and Rehabilitation Sciences, James Cook University, Townsville, Queensland, Australia; 2 Mogo Zoo, Mogo, New South Wales, Australia; The Ohio State University, United States of America

## Abstract

The amphibian chytrid fungus *Batrachochytrium dendrobatidis* (*Bd*) has caused mass mortality leading to population declines and extinctions in many frog species worldwide. The lack of host resistance may be due to fungal immunosuppressive effects that have been observed when *Bd* is incubated with cultured lymphocytes, but whether *in vivo* host immunosuppression occurs is unknown. We used a broad range of hematologic and protein electrophoresis biomarkers, along with various functional tests, to assess immune competence in common green (*Litoria caerulea*) and white-lipped (*L. infrafrenata*) tree frogs experimentally infected with *Bd*. Compared with uninfected frogs, *Bd* infection in *L. caerulea* caused a reduction in immunoglobulin and splenic lymphocyte responses to antigenic stimulation with sheep red blood cells, along with decreased white blood cell and serum protein concentrations, indicating possible impaired immune response capability of *Bd*-infected frogs. This is the first *in vivo* study suggesting that infection with *Bd* causes multiple defects in systemic host immune function, and this may contribute to disease development in susceptible host species. Although *L. infrafrenata* failed to maintain *Bd* infection after exposure, white blood cell and serum globulin concentrations were lower in recovered frogs compared with unexposed frogs, but antigen-specific serum and splenic antibody, and splenic cellular, responses were similar in both recovered and unexposed frogs. This may indicate potential systemic costs associated with infection clearance and/or redirection of host resources towards more effective mechanisms to overcome infection. No clear mechanism for resistance was identified in *L. infrafrenata*, suggesting that localized and/or innate immune defense mechanisms may be important factors involved in disease resistance in this species.

## Introduction

The recent global spread of the emerging infectious disease chytridiomycosis has caused declines and extinctions of many amphibian species [1]–[3]. The causative fungal skin pathogen, *Batrachochytrium dendrobatidis* (*Bd*), has had the most devastating impact in remote and protected mountainous regions, where abundant populations crashed within months of its arrival [1], [2], [4]. Environmental changes such as pollution and climate are generally not considered primary factors in its emergence – *Bd* can clearly cause high mortality rates in healthy, immune competent populations [3]. Combined with its ability to spread rapidly through host populations and persist even at low host densities, it has had an unprecedented effect on amphibian biodiversity [1], [2], [5]–[7]. If naïve susceptible amphibian populations survive introduction of *Bd*, it becomes endemic with reduced mortality rates and sometimes partial recovery, suggesting selection for host resistance and/or waning pathogen virulence [8].

Morbidity and mortality rates in post-metamorphic amphibians vary greatly among species and can reach up to 100% in susceptible captive anuran species, including the common green tree frog (*Litoria caerulea*) [1], [9]–[12]. Fatal pathophysiological changes include epidermal degeneration, inhibited epidermal electrolyte transport, systemic electrolyte disturbances (hyponatremia and hypokalemia) [11]–[13], severe hypovolemia secondary to dehydration [13] and asystolic cardiac arrest [12].

Wide variation in susceptibility to chytridiomycosis exists between species, populations and individuals. Within a species or population, local environmental conditions and specific behavioural characteristics can influence disease dynamics; *Bd* is susceptible to heat and desiccation, and frogs inhabiting unfavourable habitats have improved survival [8], [10], [14], [15]. Although recent progress has been made in understanding aspects of resistance to *Bd*, the mechanisms of immunity appear complex and much remains unknown. The post-metamorphic amphibian immune system is fundamentally similar to that of mammals, demonstrating innate and adaptive responses including specific cell-mediated and antibody responses, and immunoglobulin isotype heterogeneity [16]–[19]. Innate host defense mechanisms, such as antimicrobial skin peptides and symbiotic bacteria, may influence susceptibility to *Bd* infection [20], [21].

To date, little evidence of an effective localized or systemic adaptive immune response in *Bd*-infected *Rana*, *Silurana* or *Litoria* species has been found [9], [21]–[24]. Activation of innate and adaptive immunity has recently been suggested to be an important component of natural *Bd* resistance in *Xenopus laevis* [25]. Knowledge of amphibian immune responses to fungal pathogens is extremely limited and the contrasting findings of the few studies available highlight the need to broaden the taxonomic focus of future immunologic studies [23].

Furthermore, there is a critical knowledge gap about why *Bd*-susceptible amphibians fail to mount an effective immune response: is it due to pathogen immune evasion, host immunosuppression or a combination of the two? Negligible cellular inflammation occurs in the skin of infected frogs, suggesting sporangia may evade host immune recognition due to their intracellular location within the superficial epidermis [26]. Recent genetic, stress hormone and *in vitro* immune function studies indirectly suggest *Bd* may actively suppress the host immune response [27]–[31].

The overall aim of our study was to determine whether *Bd* infection suppresses systemic innate and adaptive host immune responses. We used diverse methods, previously established in mammals and birds, to study immune structure and function in *Bd*-infected and control frogs. Methodology involved measuring 1) mass and cellularity of immune organs, 2) total and differential peripheral white blood cell (WBC) counts, 3) serum protein fraction concentrations via gel electrophoresis, 4) *in vivo* phytohemagglutinin (PHA) skin response, and 5) *in vivo* anti-sheep red blood cell (SRBC) antibody response. Immunization with SRBC to evaluate humoral immunity via serum and splenic hemolytic antibody production has been previously reported in three anuran species: *Rana pipiens, Bufo arenarum* and *X. laevis* [32]–[35]. The T-cell mitogen PHA has been used to evaluate anuran splenocyte, thymocyte and lymphocyte proliferative responses *in vitro* [31], [36]–[38]. Only two reports describe the *in vivo* PHA skin response test to assess cell-mediated immunity in adult anurans [39], [40], and apart from assessment of skin peptide profiles [41], there are no reports describing innate or adaptive immunity in *Litoria* species. Our results showed that all of the methods, with the exception of the PHA skin test, were reliable for assessing immune function in the species studied, and that chronic *Bd* infection in *L. caerulea* caused multiple systemic immune function defects. *Litoria infrafrenata* failed to maintain infection with *Bd* after experimental exposure, but recovered frogs had lower white blood cell and serum globulin responses compared with unexposed frogs, suggesting potential costs associated with infection clearance and/or redirection of host resources towards more effective mechanisms to combat infection.

## Materials and Methods

### Ethics Statement

This study was carried out in strict accordance with the recommendations in the Australian Code of Practice for the Care and Use of Animals for Scientific Purposes of the National Health and Medical Research Council. The research protocols were approved by the James Cook University Animal Ethics Committee (A1085) and the Queensland Parks and Wildlife Service (Scientific Purposes Permit WISP03866106). All blood sampling and initiation of *in vivo* tests were performed under tricaine methanesulfonate general anaesthesia; frogs were euthanized at the end of the study by cardiac exsanguination following tricaine methanesulfonate general anaesthesia; all efforts were made to minimise suffering throughout the study. All field locations and activities, including collection of frogs from the wild, were approved by the Queensland Parks and Wildlife Service (Scientific Purposes Permit WISP03866106).

### Animals

Free-ranging clinically healthy adult individuals of the common green tree frog (*L. caerulea*, n = 20) and the white-lipped tree frog (*L. infrafrenata*, n = 20) were collected from widespread residential and semi-rural areas in and around Cairns and Townsville in far northern Queensland, Australia. The two species were selected based on their large body size, relative ease of capture, wide distribution and stable conservation status. Each frog was placed, using a new powder-free nitrile glove, into an individual plastic holding container (70×95×150 mm^3^) for transport. Frogs were housed in individual plastic containers (230×230×350 mm^3^) in temperature (20–22°C) and light (12L/12D) controlled quarantine facilities at James Cook University (Cairns). Aged tap water was changed daily and frogs were fed large domestic crickets (*Acheta domestica*) dusted with superfine calcium carbonate (Cattlekare, Dandenong, VIC) and multivitamin powder (Reptivite, Zoo Med Laboratories Inc., San Luis Obispo, CA), ad libitum each day.

Frogs from each species were randomly assigned equally between two experimental trials. Before the trials commenced, each frog was clinically examined by a veterinarian, weighed, and a swab sample was collected from the ventral skin surfaces for determination of *Bd* zoospore equivalents by real-time polymerase chain reaction (PCR) analysis (James Cook University, Townsville) [42]. All swab samples were analyzed in triplicate and compared with James Cook University zoospore standards. All frogs were negative for *Bd* prior to commencement of the experimental trials.

### Experimental Design 1: Uninfected Tree Frogs

This was designed as a pilot study to validate functional immune tests in healthy frogs from the two species. At the start of Experiment 1 (day 0), *L. caerulea* (n = 10) and *L. infrafrenata* (n = 10) were anesthetized for sampling for general immunological and hematological biomarkers and for initiation of functional tests for immune competence. Anesthesia was induced by shallow immersion in 0.20% (*L. infrafrenata*) or 0.25% (*L. caerulea*) ethyl 3-aminobenzoate methanesulfonic acid solution (tricaine methanesulfonate, Sigma-Aldrich Inc., St Louis, MO) buffered with 10 mEql^−1^ sodium bicarbonate solution (8.4%, Pro Care Animal Health, Dandenong, VIC).

Blood samples (250–500 µl, <1% body weight) were collected for hematologic, plasma biochemical and serum protein electrophoretic analysis from dorsally recumbent frogs via cardiocentesis with a 1 ml syringe and 25 g needle (Terumo Corporation, Binan, Laguna). The PHA skin and the SRBC antibody response tests were also initiated at this time.

On day 7, each frog was euthanized by cardiac exsanguination following induction of anesthesia as previously described. Blood samples were collected for hematologic, plasma biochemical and serum protein electrophoretic analysis and for SRBC antibody assay. Spleen, liver and kidneys were dissected, weighed and recorded as % body weight. The spleen was immediately processed for determination of total lymphocyte count, cell viability and rosette formation by antibody-producing cells. These measurements are described below.

### Experimental Design 2: *Bd*-infected Tree Frogs

At the start of Experiment 2 (day 0), *L. caerulea* (n = 10) and *L. infrafrenata* (n = 10) were anesthetized and blood samples were collected for analysis as described in Experiment 1. Frogs were then exposed to *Bd* via shallow immersion in a 25 ml bath of dilute electrolyte solution (mmol l^−1^: KH_2_PO_4_ 1, CaCl_2_.H_2_O 0.2, MgCl_2_ 0.1) inoculated with 250,000 zoospores for 24 h, after which they were returned to their holding containers with aged tap water. During the exposure period, frogs were held in small individual plastic containers (50×100×150 mm^3^) with a lid to ensure continuous contact of the ventral skin surfaces with the inoculum.

Frogs were weighed and swabs collected for PCR at 10, 20, 30, 40, 50, 60, 75 and 82 d post-exposure. On day 30 post-exposure, frogs were anesthetized and blood samples collected for hematologic and plasma biochemical analysis. On day 75 post-exposure (corresponding to day 0 in the uninfected frogs from Experiment 1), *Bd*-exposed frogs were again anesthetized for blood sampling and for initiation of the PHA skin and SRBC antibody response tests. One *Bd*-exposed *L. infrafrenata* failed to recover post-anesthesia and was excluded from the trial. On day 82 post-exposure (corresponding to day 7 in the uninfected frogs from Experiment 1), each *Bd*-exposed frog (*L. caerulea* n = 10, *L. infrafrenata* n = 9) was euthanized by cardiac exsanguination following anesthesia. Blood, spleen, liver and kidney samples were collected as per Experiment 1. This time point for testing was chosen to ensure that frogs infected early in the trial maintained infection, but had not yet developed severe clinical signs of disease which would confound our immune function assays.

### Hematologic and Plasma Biochemical Analysis

Blood samples from each frog were processed according to standard amphibian procedures [43], [44]. Fresh blood smears were air dried and fixed with 100% methanol; 200 µl was collected into a 0.6 ml Microtainer pediatric lithium heparin tube (Becton and Dickinson, Franklin Lakes, New Jersey); and 150–200 µl was collected into a plain 1.0 ml microcentrifuge tube (Eppendorf AG, Hamburg), centrifuged (5,590×*g* for 10 min) and the supernatant decanted and refrigerated at 4°C until submission for serum protein electrophoresis. Additional blood (500–1000 µl) from the final sample on day 7 (healthy uninfected frogs, Experiment 1) and day 82 (*Bd*-exposed frogs, Experiment 2) was collected into a plain 1.0 ml microcentrifuge tube, allowed to clot at room temperature for 1 h, then centrifuged (5,590×*g* for 10 min) and the supernatant decanted and frozen at −70°C for later SRBC antibody microtiter assay.

Total red blood cells (RBC), WBC and thrombocytes were counted manually in a modified Neubauer hemocytometer at 400× magnification with Natt-Herrick's solution as the diluent [43]–[45]. Differential WBC and polychromatophilic RBC were counted at 1000× magnification from Wright's-stained (Clinipure Wright's Stain and Wright's Buffer Concentrate, HD Scientific Supplies Pty Ltd, Wetherill Park, NSW) blood smears. Well-mixed whole blood (5 µl) was drawn into a pediatric microhematocrit tube (Becton and Dickinson, Franklin Lakes, NJ) and centrifuged (112×*g* for 2 min) for packed cell volume (PCV) measurement. Hemoglobin (Hb) was assayed manually using the cyanomethemoglobin method modified for species with nucleated RBC [46], [47] and specifically for amphibians [44]. Mean corpuscular volume (MCV), mean corpuscular Hb (MCH) and MCH concentration (MCHC) were calculated from Hb, PCV and RBC values using standard formulae [48].

Plasma biochemical analytes were measured from 100 µl of whole blood using the automated bench-top VetScan VS2 Chemistry Analyzer and VetScan Avian/Reptilian Profile Plus rotor (Abaxis Inc., Union City, CA) and included: aspartate aminotransferase (AST), uric acid (UA), creatine kinase (CK), glucose, calcium, phosphorus, potassium and sodium.

### Serum Protein Electrophoresis

Serum samples (n = 97) were submitted to a commercial reference laboratory (Gribbles Veterinary Pathology, Clayton, VIC) for determination of total protein and protein fraction (albumin, total globulins and α-1, α-2, β and γ globulin) concentrations. Electrophoresis was conducted according to the manufacturer's recommendations using the semi-automated agarose gel electrophoresis system (Hydrasys, Sebia Inc., Norcross, GA) and the split protein β1 and β2 gel reagent (Hydragel 30, Sebia Inc., Norcross, GA). The resultant gel was fixed, stained and scanned using the same equipment. Densitometer laser tracings were used to measure protein fraction percentages [49], and absolute values were determined on the basis of biuret total protein measurement. The albumin-globulin (A–G) ratio was calculated by dividing the albumin value by the sum of the globulin fraction values.

### PHA Skin Response Test

The PHA skin response test for T-cell mediated immunity was initiated following standard avian and mammalian procedures [50]–[52] adapted for anurans. A 0.1 ml dose of 0.5% PHA-P (Sigma-Aldrich Inc., St Louis, MO) in phosphate-buffered saline (PBS) (pH 7.4, Sigma-Aldrich Inc., St Louis, MO) was injected intradermally in the interdigital webbing of the left hind foot between the second and third phalanges with a 1 ml syringe and 27 g needle. The same volume of PBS was injected intradermally as a control in the right hind foot interdigital webbing. The thickness of each injection site was measured to the nearest 0.02 mm using manual vernier callipers (Mitutoyo Corporation, Kanagawa) immediately before and then 6, 12, 24 and 48 h post-injection. The PHA stimulation response was calculated as the change in the thickness (mm) of the PHA-injected interdigital site minus the change in thickness of the control site.

### Serum SRBC Antibody Assay

The SRBC antibody response test was initiated following standard avian and mammalian procedures [51], [53] adapted for anurans. Each frog was injected intracelomically with 0.5 ml of a 10% suspension of SRBC (Sigma-Aldrich Inc., St Louis, MO) in PBS with a 1 ml syringe and 23 g needle.

Total (IgM and IgY) and 2-mercaptoethanol-resistant (IgY) SRBC antibody activities were measured 7 d post-immunization using a standard microtiter method [52], [53]. Saline (PBS, 50 µl) was added to each well in 96-well round-bottomed microtiter plates (Eppendorf AG, Hamburg). Serum (50 µl) was added to the first well of each row, and serial two-fold dilutions were performed across rows. Fifty µl of 0.5% SRBC suspension in PBS was then added to each well and the plates incubated at 37°C for 3 h and then overnight at room temperature. Titers were recorded as LOG_10_ of the reciprocal of the highest dilution showing agglutination. To measure IgY titers, serum samples were incubated for 60 min with 0.2 M 2-mercaptoethanol (Sigma-Aldrich Inc., St Louis, MO) before dilution. All serum samples were assayed in duplicate and the same batch of SRBC was used for all immunizations and assays.

### Splenic Lymphocyte Count and Cell Viability Determination

Each spleen was divided equally by weight and processed following standard avian and mammalian procedures [51]–[53]. One half was fixed in 10% neutral buffered formalin, the other was homogenised into a single cell suspension in 1.0 ml Hanks balanced salt solution (HBSS) (Sigma-Aldrich Inc., St Louis, MO) with a scalpel blade. Total splenic lymphocytes in the cell suspension were counted manually in a modified Neubauer hemocytometer at 400× magnification.

Spleen cell viability was determined by incubating 0.2 ml spleen cell suspension with 0.3 ml HBSS and 0.5 ml trypan blue solution (Sigma-Aldrich Inc., St Louis, MO) for 10 min [54], [55]. Stained (non-viable) and unstained (viable) cells were counted manually in a modified Neubauer hemocytometer at 400× magnification. Cell viability % was calculated by dividing the mean number of unstained cells by the mean total number of stained and unstained cells.

### Splenic Rosette-forming Cell Assay

This method was adapted from a combination of techniques previously described for rosette-forming [56], [57] and plaque-forming [58], [59] cell assays. Rosette-forming IgM-producing splenic lymphocytes sensitized to SRBC were counted in two monolayer chambers following incubation of 0.04 ml spleen single cell suspension with 0.2 ml HBSS and 0.2 ml 10% SRBC suspension at 37°C for 1 h. Rosette-forming cells (RFC) were counted manually at 50× magnification and the total number of RFC calculated per spleen.

### Culture and harvest of *Bd*

The *Bd* isolate (Melbourne-L.lesueuri-00-LB-1-p19) was originally harvested from a clinically diseased captive juvenile *L. lesueuri* and cultured on tryptone/gelatin hydrolysate/lactose (TGhL) agar with streptomycin sulfate and benzylpenicillin (Sigma Aldrich Inc., St Louis, MO) [60]. Cultures were maintained in half-strength TGhL broth at 4°C. Zoospores for frog inoculation were harvested by flooding 4 d old agar plate cultures maintained at 22°C with a dilute electrolyte solution (mmol l^−1^: KH_2_PO_4_ 1, CaCl_2_.H_2_O 0.2, MgCl_2_ 0.1) and counted in a hemocytometer (Brand GMBH and CO KG, Wertheim) [9], [61].

### Statistical Analysis

Independent-samples *t*-tests were used to compare functional tests for immune competence in healthy frogs between the two species, between healthy and *Bd*-exposed frogs within each of the two species, and between infected *L. caerulea* with low *Bd* loads (<1,000 zoospores) and high *Bd* loads (>1,000 zoospores). Variables analyzed included skin PHA stimulation, serum IgY and combined serum IgM/IgY titers, total splenic cell count, splenic RFC count, splenic cell viability, ratios of kidney, liver and spleen to body weight, and various hematologic, plasma biochemical and protein electrophoretic parameters.

Paired-samples *t*-tests were used to compare various hematologic, plasma biochemical and protein electrophoretic variables pre- and post-immune stimulation within each experimental group (healthy and *Bd*-exposed) for each species.

The software package PASW Statistics (Version 18, 2009, SPSS Inc., Chicago, IL) was used for all analyses, and statistical significance was set at ≤0.050 in all cases.

## Results

### Bd-infected *Litoria caerulea*

At day 75 post-exposure when immune function tests were initiated, 100% of exposed *L. caerulea* (10/10) tested positive for *Bd* on PCR. Zoospore counts per sample ranged from 24 to >10,000; six frogs had low *Bd* loads (<1,000 zoospores) and four had high *Bd* loads (>1,000 zoospores). One frog with >10,000 zoospores showed mild clinical signs of disease including lethargy and cutaneous erythema; all other frogs were clinically normal.

Mean splenic total lymphocyte and RFC counts, splenic cell viability and liver-body weight ratio post-immune stimulation were lower in *Bd*-infected *L. caerulea* (n = 10) compared with the uninfected frogs (n = 10) (Table 1). Mean responses to all of the functional tests for immune competence did not differ between infected frogs with low and high *Bd* loads (*P*>0.050 in all cases).

**Table 1:** Functional test results for immune competence in uninfected and *Batrachochytrium dendrobatidis*-exposed *Litoria caerulea* and *L. infrafrenata* following stimulation with intradermal phytohemagglutinin (PHA) and intracelomic sheep red blood cells.

Species	*Litoria caerulea*					*Litoria infrafrenata*				
Status	Healthy (n = 10)		Infected^a^ (n = 10)			Healthy (n = 10)		Exposed^b^ (n = 9)		
Immune Parameter	Mean	SD	Mean	SD	*P* value	Mean	SD	Mean	SD	*P* value
Skin PHA Stimulation (mm)	0.14	0.46	0.04	0.08	0.495	0.25	0.39	−0.19	0.34	0.017
IgM + IgY Titre (LOG_10_)	4.7	3.1	3.7	1.3	0.370	4.9	2.3	4.6	2.7	0.800
IgY Titre (LOG_10_)	3.4^c^	2.5	4.1	4.2	0.529	4.0^c^	2.6	6.5	3.6	0.062
Splenic Cell Count (x10^6^)	36.4	14.9	12.7	8.6	0.000	40.6	14.4	31.4	21.0	0.277
Splenic Cell Viability (%)	69.0	11.5	35.7	14.5	0.000	70.4	11.4	62.0	6.8	0.070
Rosette-forming Cells (x10^3^)	2423	1369	898	629	0.005	855	497	755	356	0.623
Final BW^d^ (g)	41.5	19.1	59.8	12.8	-	37.2	12.1	66.9	13.4	-
Kidney-BW Ratio	0.60	0.13	0.51	0.16	0.164	0.64	0.13	0.51	0.06	0.011
Liver-BW Ratio	5.23	1.61	2.89	0.64	0.001	3.23	0.65	3.39	1.16	0.726
Spleen-BW Ratio	0.04	0.01	0.04	0.01	0.216	0.13	0.07	0.12	0.06	0.555

a 100% of 10 exposed *L. caerulea* were infected 75 d post-exposure to *Bd*.

b 0% of 9 exposed *L. infrafrenata* were infected 75 d post-exposure to *Bd*.

c n = 9.

Following immune stimulation of *Bd*-infected *L. caerulea*, mean thrombocyte count decreased and A-G ratio increased; all other hematologic, plasma biochemical and serum protein electrophoretic parameters did not differ significantly pre- and post-stimulation (Tables 2 and 3).

**Table 2:** Pre- and post-immune stimulation hematologic values for uninfected and *Batrachochytrium dendrobatidis*-infected *Litoria caerulea*.

Status	Uninfected (n = 10)					Infected (n = 10)					
Immune Stimulation	Pre (day 0)		Post (day 7)			Pre (day 0)		Post (day 7)			
Parameter	Mean	SD	Mean	SD	*P* value^a^	Mean	SD	Mean	SD	*P* value^a^	*P* value^b^
PCV (%)	35.9	3.4	28.9	4.8	0.004	36.1	4.0	34.5	7.5	0.485	0.074
Hb (g dl^−1^)	8.7	1.2	6.2	1.4	0.000	9.0	2.4	8.6	1.8	0.670	0.042
RBC (x10^9^ l^−1^)	694	135	588	120	0.137	553	90	580	132	0.515	0.098
MCV (fl)	536	117	503	107	0.402	663	91	606	107	0.237	0.697
MCH (pg)	129	25	107	24	0.021	165	44	151	26	0.493	0.675
MCHC (g l^−1^)	245	38	214	30	0.010	247	46	252	33	0.827	0.137
Thrombocyte (×10^9^ l^−1^)	34.8	9.0	30.5	7.3	0.299	27.0	5.9	23.6	4.0	0.042	0.823
WBC (x10^9^ l^−1^)	24.6	8.9	39.7	15.4	0.026	6.8	1.9	8.5	4.0	0.175	0.044
Neutrophil (×10^9^ l^−1^)	3.9	1.8	4.9	2.2	0.303	2.0	1.0	2.3	1.8	0.576	0.513
Lymphocyte (×10^9^ l^−1^)	19.0	7.1	31.2	13.9	0.026	4.1	1.5	5.0	1.9	0.179	0.035
Neut-lymph ratio	0.22	0.10	0.18	0.09	0.224	0.54	0.32	0.46	0.24	0.376	0.694
Monocyte (×10^9^ l^−1^)	1.3	0.9	3.1	1.7	0.005	0.6	0.7	1.1	0.7	0.115	0.038
Eosinophil (×10^9^ l^−1^)	0.50	0.95	0.50	0.68	0.814	0.06	0.08	0.10	0.12	0.055	0.676
Basophil (×10^9^ l^−1^)	0.00	0.00	0.04	0.12	0.343	0.00	0.00	0.00	0.00	-	0.343
Polychromasia (%)	6.9	4.0	13.8	10.5	0.044	3.3	1.8	2.6	2.8	0.396	0.032

d BW, body weight.

a Paired-samples *t*-tests between days 0 and 7 within each group (uninfected and infected).

b Independent-samples *t*-tests between the two groups for the *change* in each variable from day 0 to day 7.

**Table 3:** Pre- and post-immune stimulation plasma biochemical and serum protein electrophoretic values for uninfected and *Batrachochytrium dendrobatidis*-infected *Litoria caerulea*.

Status	Uninfected (n = 10)					Infected (n = 10)					
Immune Stimulation	Pre (day 0)		Post (day 7)			Pre (day 0)		Post (day 7)			
Parameter	Mean	SD	Mean	SD	*P* value^a^	Mean	SD	Mean	SD	*P* value^a^	*P* value^b^
AST (U l^−1^)	69	37	73	34	0.800	68	14	97	42	0.068	0.268
CK (U l^−1^)	495	344	636	496	0.566	282	185	555	337	0.074	0.632
Uric Acid (µmol l^−1^)	37	27	40	22	0.813	35	18	37	29	0.811	0.937
Glucose (mmol l^−1^)	3.5	0.8	3.4	0.7	0.766	4.5	0.9	4.6	0.9	0.715	0.640
Calcium (mmol l^−1^)	3.03	0.46	3.00	0.54	0.861	2.75	0.40	2.72	0.40	0.697	0.936
Phosphorus (mmol l^−1^)	1.70	0.46	1.49	0.42	0.194	0.88	0.23	0.83	0.35	0.587	0.389
Ca-P ratio	1.89	0.58	2.16	0.59	0.193	3.29	0.82	3.78	1.42	0.260	0.639
Potassium (mmol l^−1^)	4.8	1.7	4.2	1.9	0.288	5.2	1.5	4.2	2.2	0.343	0.749
Sodium (mmol l^−1^)	111.7	6.1	111.5	4.8	0.929	109.5	6.2	108.5	4.9	0.535	0.768
Total Protein (g l^−1^)	57.4	6.3	62.6	6.8	0.106	57	7	54	6	0.353	0.069
A-G ratio	0.57	0.12	0.44	0.09	0.008	0.46	0.16	0.54	0.17	0.000	0.000
Albumin (g l^−1^)	20.5	3.4	18.7	2.2	0.130	18	5	18	4	0.604	0.106
Total globulins (g l^−1^)	36.9	5.2	43.9	6.6	0.024	39.2	4.9	35.8	6.6	0.207	0.010
α-1 globulin (g l^−1^)	22.7	4.1	28.0	4.0	0.026	21.5	3.6	20.7	5.2	0.620	0.030
α-2 globulin (g l^−1^)	6.9	1.2	8.2	3.1	0.302	9.2	1.5	7.9	2.0	0.092	0.072
β globulin (g l^−1^)	5.2	1.6	4.9	1.5	0.696	5.8	1.7	5.0	1.0	0.077	0.541
γ globulin (g l^−1^)	2.2	0.8	2.8	1.1	0.023	2.7	1.0	2.2	0.7	0.072	0.004

a Paired-samples *t*-tests between days 0 and 7 within each group (uninfected and infected).

b Independent-samples *t*-tests between the two groups for the *change* in each variable from day 0 to day 7.

**Figure 1:**
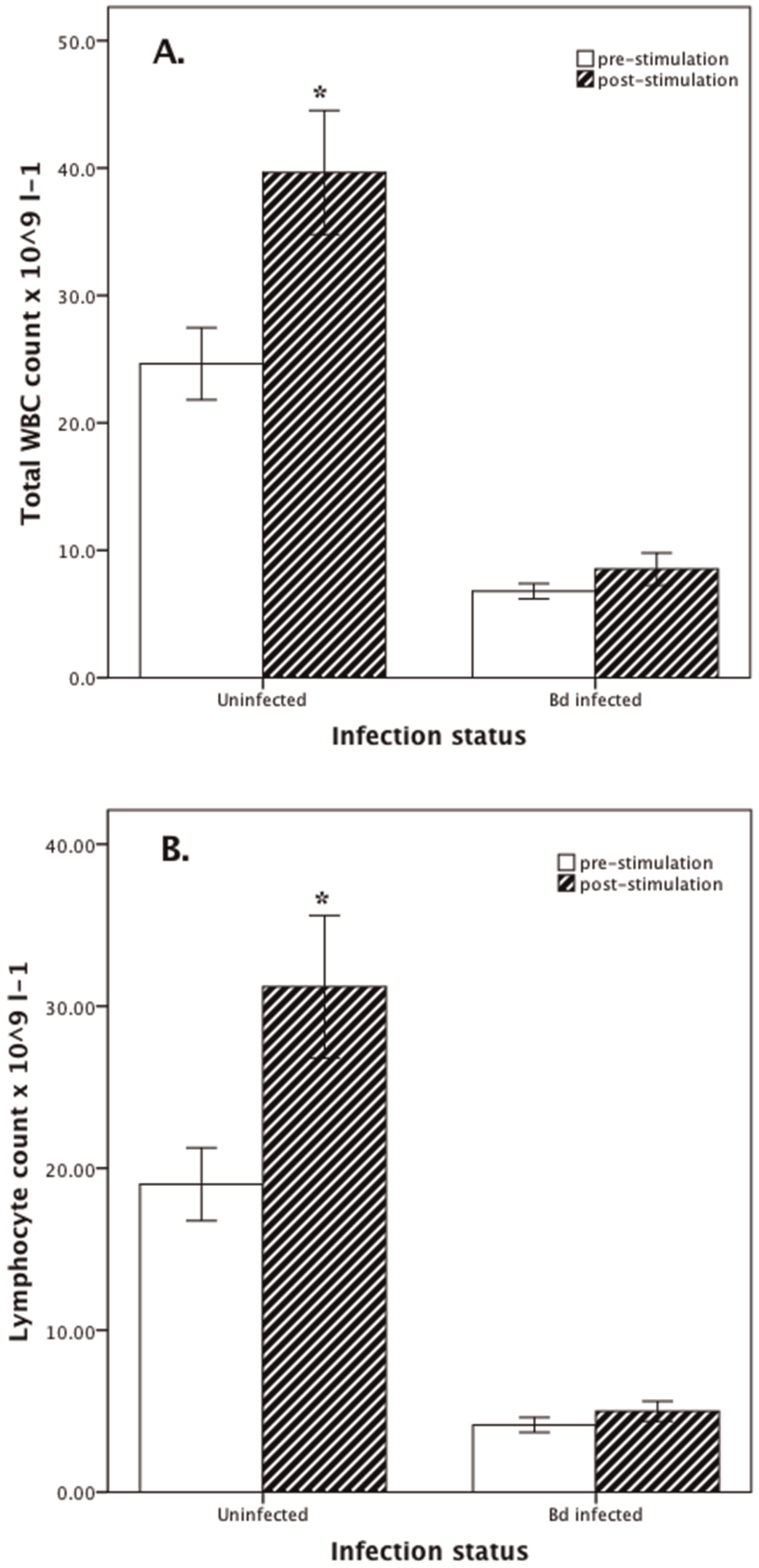
White blood cell counts of *Litoria caerulea*. Total white blood cell (WBC) (Fig. 1A) and lymphocyte (Fig. 1B) counts (x10^9^ l^−1^) pre- and post-immune stimulation in healthy uninfected (n = 10) and *Batrachochytrium dendrobatidis*-infected (n = 10) *Litoria caerulea*. Bars are mean ± SEM. *P<0.050 within each group.

Compared with *Bd*-infected individuals, immune stimulation of healthy uninfected *L. caerulea* caused a greater magnitude of increase in total WBC (M+15.0 (SD 17.8) versus +1.7 (3.7) ×10^9^ l^−1^) (Fig. 1A), lymphocyte (M+12.2 (SD 14.5) versus +0.9 (1.8) ×10^9^ l^−1^) (Fig. 1B) and monocyte (M+1.8 (SD 1.5) versus +0.5 (0.9) ×10^9^ l^−1^) counts and polychromasia (M+6.9 (SD 9.3) versus −0.7 (2.5) %), and a greater decrease in Hb concentration (M −2.6 (SD 1.2) versus −0.4 (2.9) g dl^−1^) (Table 2).

**Figure 2:**
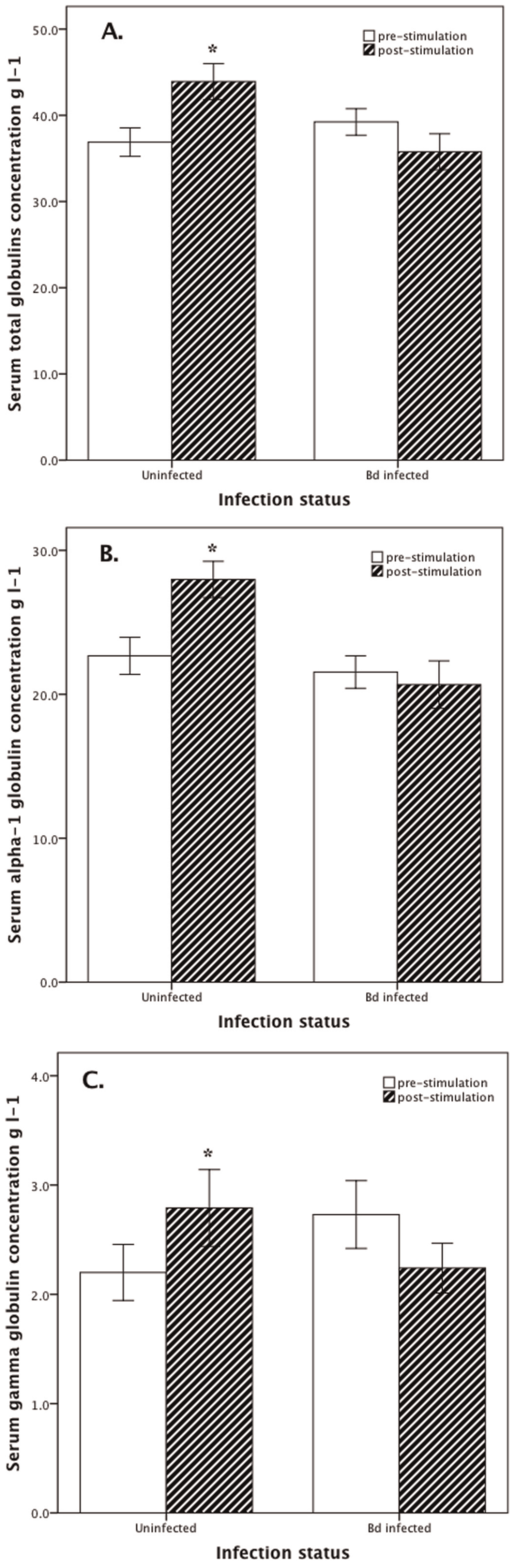
Serum globulin fractions of *Litoria caerulea*. Serum total globulins (Fig. 2A), α-1 globulin (Fig. 2B) and γ globulin (Fig. 2C) concentrations (g l^−1^) pre- and post-immune stimulation in healthy uninfected (n = 10) and *Batrachochytrium dendrobatidis*-infected (n = 10) *Litoria caerulea*. Bars are mean ± SEM. *P<0.050 within each group.

Compared with *Bd*-infected individuals, immune stimulation of healthy uninfected *L. caerulea* caused a significantly greater increase in total globulins (M+7.0 (SD 8.2) versus −3.5 (8.1) g/L) (Fig. 2A), α-1 globulin (M+5.3 (SD 6.3) versus −0.9 (5.4) g l^−1^) (Fig. 2B) and γ globulin (M+0.6 (SD 0.7) versus −0.5 (0.8) g l^−1^) (Fig. 2C) concentrations, and a greater decrease in A-G ratio (M −0.13 (SD 0.12) versus +0.08 (0.03)) (Table 3).

### *Bd*-infected *Litoria infrafrenata*

At day 75 post-exposure, 0% of the nine exposed *L. infrafrenata* tested positive for *Bd* on PCR. Five frogs had low *Bd* loads at either day 10 (n = 3) or 20 (n = 2) post-exposure, but all of these were negative from day 30 onwards post-exposure, and the other four frogs tested negative throughout the experiment.

Mean skin PHA response and kidney-body weight ratio were lower in exposed uninfected *L. infrafrenata* compared with healthy uninfected individuals post-immune stimulation; mean responses to the other immune function tests did not differ between the two groups (Table 1).

Following immune stimulation of *Bd*-exposed uninfected *L. infrafrenata*, mean Hb concentration and RBC and thrombocyte counts decreased, while glucose concentration increased; all other hematologic, plasma biochemical and serum protein electrophoretic parameters did not differ significantly (Tables 4 and 5).

**Table 4:** Pre- and post-immune stimulation hematologic values for uninfected and *Batrachochytrium dendrobatidis*-exposed but uninfected *Litoria infrafrenata*.

Status	Uninfected (n = 10)					Exposed uninfected (n = 9)					
Immune Stimulation	Pre (day 0)		Post (day 7)			Pre (day 0)		Post (day 7)			
Parameter	Mean	SD	Mean	SD	*P* value^a^	Mean	SD	Mean	SD	*P* value^a^	*P* value^b^
PCV (%)	26.9	7.0	25.4	4.1	0.548	35.7	6.0	32.3	4.8	0.079	0.548
Hb (g d l^−1^)	7.1	2.5	5.6	1.1	0.086	8.6	1.3	7.4	1.7	0.024	0.691
RBC (x10^9^ l^−1^)	736	208	630	129	0.048	783	109	662	108	0.018	0.811
MCV (fl)	383	155	421	124	0.450	459	74	500	121	0.377	0.969
MCH (pg)	107	60	93	32	0.525	111	19	116	38	0.674	0.457
MCHC (g l^−1^)	291	126	220	29	0.142	245	41	232	47	0.306	0.240
Thrombocyte (×10^9^ l^−1^)	54.8	12.9	27.2	7.2	0.000	33.7	10.1	26.8	7.0	0.036	0.001
WBC (x10^9^ l^−1^)	32.9	8.7	47.6	10.2	0.005	33.7	27.0	31.0	17.4	0.616	0.015
Neutrophil (×10^9^ l^−1^)	5.8	5.9	16.4	5.1	0.002	12.1	14.3	9.3	10.0	0.204	0.001
Lymphocyte (×10^9^ l^−1^)	24.3	7.7	25.5	6.2	0.726	17.8	11.1	17.3	11.1	0.860	0.700
Neut-lymph ratio	0.28	0.36	0.67	0.24	0.038	0.66	0.47	0.71	0.71	0.726	0.120
Monocyte (×10^9^ l^−1^)	2.1	1.4	5.6	3.5	0.023	3.3	3.9	4.0	3.1	0.522	0.131
Eosinophil (×10^9^ l^−1^)	0.25	0.44	0.04	0.13	0.205	0.57	0.76	0.34	0.45	0.355	0.925
Basophil (×10^9^ l^−1^)	0.47	0.79	0.00	0.00	0.095	0.00	0.00	0.00	0.00	-	0.095
Polychromasia (%)	5.5	3.6	15.3	7.9	0.004	9.3	5.1	11.1	6.7	0.108	0.013

a Paired-samples *t*-tests between days 0 and 7 within each group (uninfected and exposed).

b Independent-samples *t*-tests between the two groups for the *change* in each variable from day 0 to day 7.

**Table 5:** Pre- and post-immune stimulation plasma biochemical and serum protein electrophoretic values for uninfected and *Batrachochytrium dendrobatidis*-exposed but uninfected *Litoria infrafrenata*.

Status	Uninfected (n = 10)					Exposed uninfected (n = 9)					
Immune Stimulation	Pre (day 0)		Post (day 7)			Pre (day 0)		Post (day 7)			
Parameter	Mean	SD	Mean	SD	*P* value^a^	Mean	SD	Mean	SD	*P* value^a^	*P* value^b^
AST (U l^−1^)	248	406	171	93	0.498	100	52	127	84	0.309	0.390
CK (U l^−1^)	556	286	1137	560	0.013	918	864	1276	929	0.218	0.539
Uric Acid (µmol l^−1^)	8	17	24	37	0.251	3.8	3.4	5.3	6.6	0.433	0.302
Glucose (mmol l^−1^)	3.6	0.6	2.9	0.8	0.036	3.4	0.4	4.2	1.0	0.020	0.002
Calcium (mmol l^−1^)	2.29	2.09	2.38	0.99	0.827	2.90	0.64	3.01	0.80	0.628	0.956
Phosphorus (mmol l^−1^)	1.54	0.63	1.73	0.76	0.404	1.59	0.43	1.52	0.32	0.412	0.279
Ca-P ratio	1.38	0.49	1.47	0.40	0.608	1.88	0.36	2.01	0.45	0.388	0.841
Potassium (mmol l^−1^)	3.7	0.8	3.8	0.9	0.591	4.8	2.0	4.2	1.5	0.183	0.145
Sodium (mmol l^−1^)	107.7	2.7	110.9	4.1	0.065	110.0	3.6	108.7	3.1	0.336	0.138
Total Protein (g l^−1^)	29	6	34	7	0.007	40	8	38	5	0.500	0.065
A-G ratio	0.11	0.04	0.13	0.04	0.033	0.21	0.06	0.23	0.07	0.175	0.609
Albumin (g l^−1^)	2.9	1.1	3.9	1.4	0.013	7.0	2.7	6.9	1.0	0.866	0.123
Total globulins (g l^−1^)	26.1	6.1	30.5	5.8	0.016	33.2	5.9	31.0	5.0	0.470	0.070
α-1 globulin (g l^−1^)	9.2	2.1	11.6	4.2	0.041	11.6	2.0	9.3	3.0	0.057	0.004
α-2 globulin (g l^−1^)	5.3	1.7	6.8	2.9	0.418	6.1	2.6	5.7	2.3	0.654	0.079
β globulin (g l^−1^)	10.1	5.5	11.0	4.0	0.192	13.7	4.2	14.6	2.7	0.665	0.968
γ globulin (g l^−1^)	1.4	0.8	1.2	0.3	0.271	1.3	0.4	1.4	0.3	0.883	0.317

a Paired-samples *t*-tests between days 0 and 7 within each group (uninfected and exposed).

b Independent-samples *t*-tests between the two groups for the *change* in each variable from day 0 to day 7.

**Figure 3:**
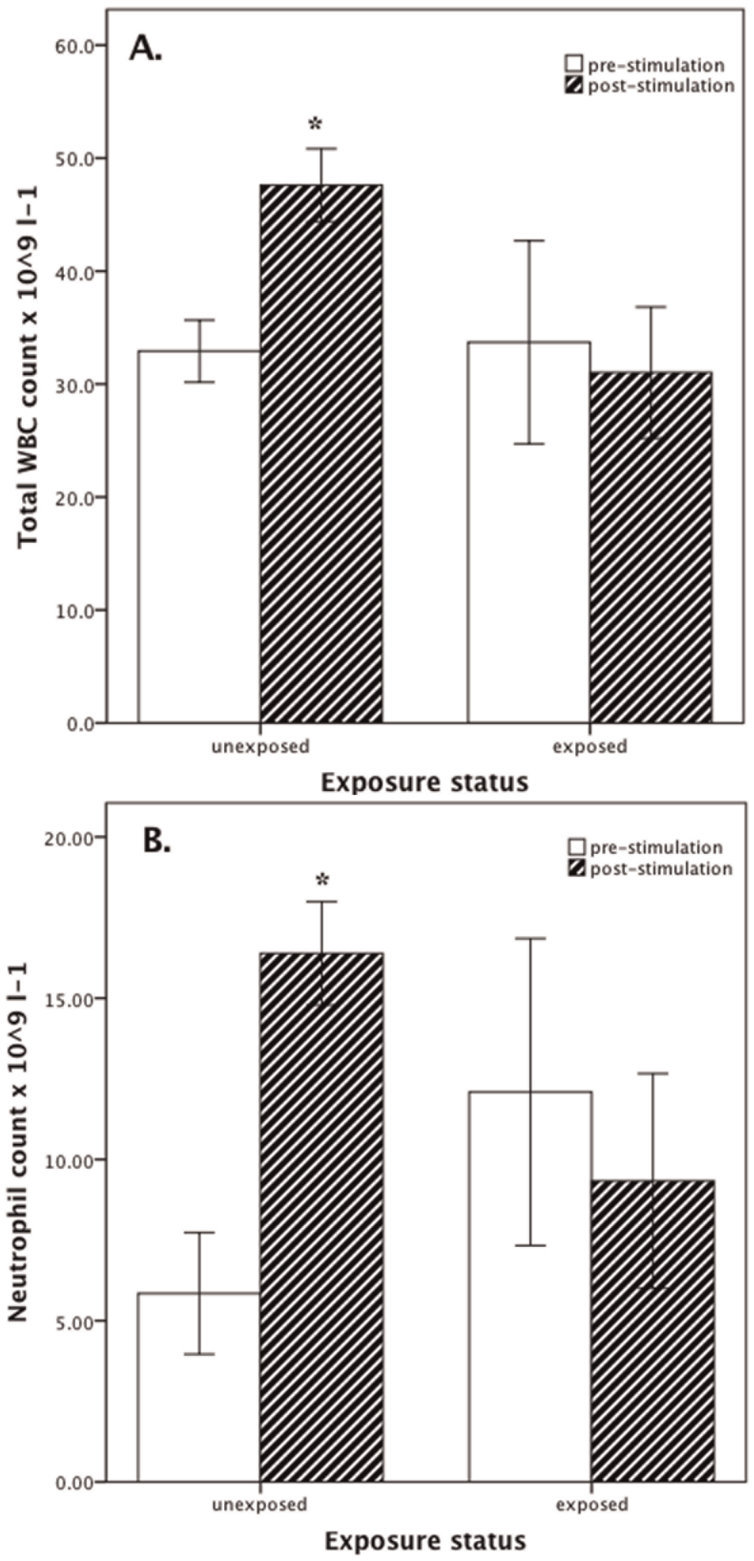
White blood cell counts of *Litoria infrafrenata*. Total white blood cell (WBC) (Fig. 3A) and neutrophil (Fig. 3B) counts (x10^9^ l^−1^) pre- and post-immune stimulation in healthy unexposed (n = 10) and *Batrachochytrium dendrobatidis*-exposed but uninfected (n = 9) *Litoria infrafrenata*. Bars are mean ± SEM. *P<0.050 within each group.

Compared with *Bd*-exposed uninfected individuals, immune stimulation of healthy uninfected *L. infrafrenata* caused a greater increase in total WBC (M+14.7 (SD 12.4) versus −2.7 (15.5) ×10^9^ l^−1^) (Fig. 3A) and neutrophil (M+10.5 (SD 7.9) versus −2.8 (6.0) ×10^9^ l^−1^) (Fig. 3B) counts and polychromasia (M+9.8 (SD 8.0) versus +1.8 (3.0) %), and a greater decrease in thrombocyte count (M −27.6 (SD 13.8) versus −6.9 (8.3) ×10^9^ l^−1^) (Table 4).

**Figure 4:**
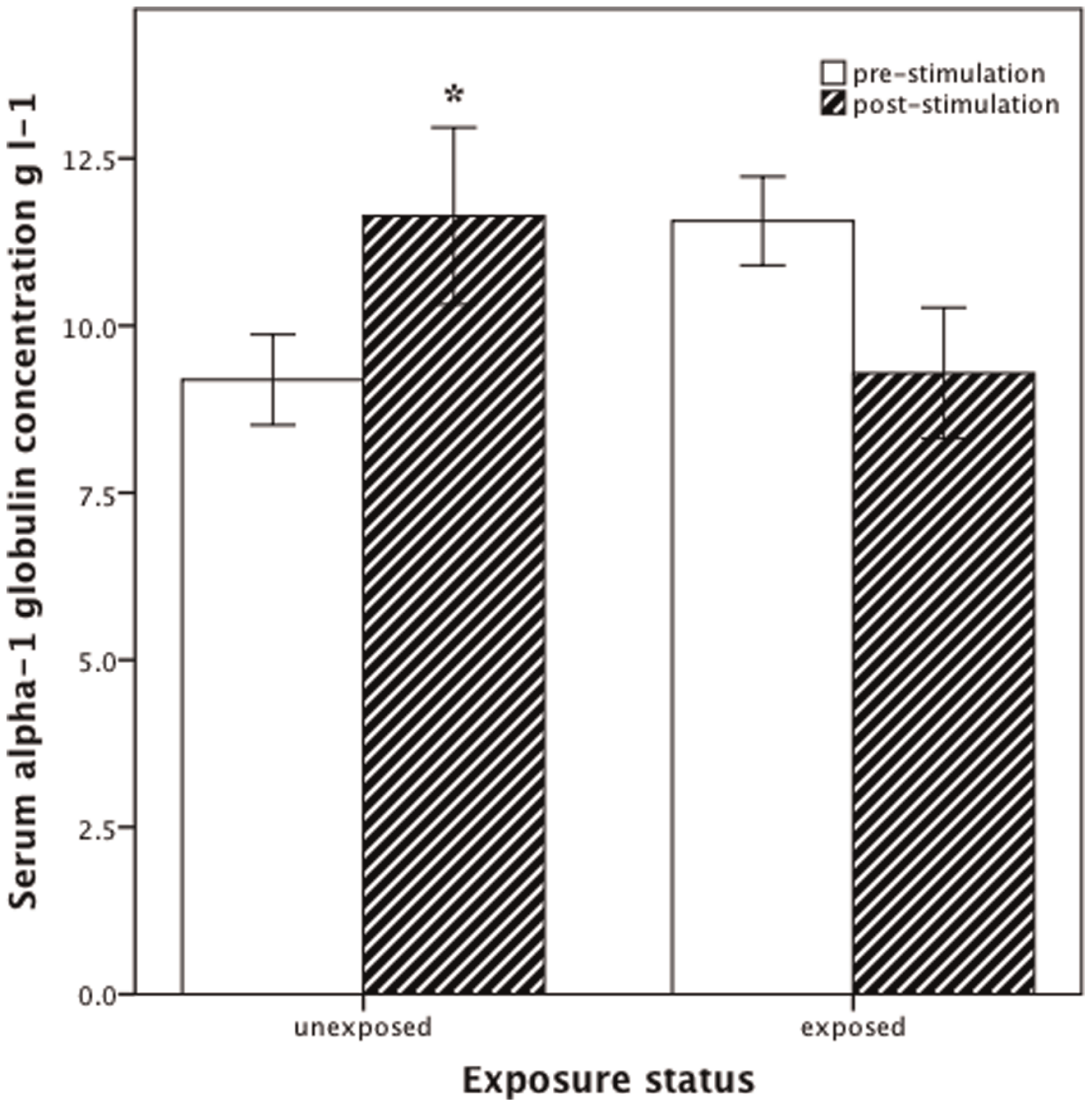
Serum α-1 globulins of *Litoria infrafrenata*. Serum α-1 globulin concentration (g l^−1^) pre- and post-immune stimulation in healthy unexposed (n = 10) and *Batrachochytrium dendrobatidis*-exposed but uninfected (n = 9) *Litoria infrafrenata*. Bars are mean ± SEM. *P<0.050 within each group.

Compared with *Bd*-exposed uninfected individuals, immune stimulation of healthy uninfected *L. infrafrenata* caused a significantly greater increase in α-1 globulin concentration (M+2.5 (SD 3.1) versus −2.3 (3.1) g l^−1^) (Fig. 4), and a greater decrease in glucose concentration (M −0.7 (SD 0.9) versus +0.7 (0.8) mmol l^−1^) (Table 5). Changes in other variables post-immune stimulation did not differ between the two groups.

### Uninfected Tree Frogs

Functional immune competence test results for uninfected *L. caerulea* and *L. infrafrenata* are presented in Table 1. Following immune stimulation, mean total splenic RFC (*P* = 0.006) and liver to body weight ratio (*P* = 0.003) were greater in *L. caerulea*, while mean spleen to body weight ratio was greater in *L. infrafrenata* (*P* = 0.001). Mean responses to the other immune function tests did not differ between the two species.

Pre- and post-immune stimulation hematologic values for uninfected *L. caerulea* and *L. infrafrenata* are presented in Tables 2 and 4 respectively. Following immune stimulation of *L. caerulea*, PCV, Hb, MCH and MCHC decreased, while total WBC, lymphocyte and monocyte counts and polychromasia % increased. In *L. infrafrenata*, total RBC and thrombocyte counts decreased following immune stimulation, while total WBC, neutrophil and monocyte counts, neutrophil-lymphocyte ratio, and polychromasia % increased.

Pre- and post-immune stimulation plasma biochemical and serum protein electrophoresis values for uninfected *L. caerulea* and *L. infrafrenata* are presented in Tables 3 and 5 respectively. Five protein fractions were defined in all samples submitted for electrophoresis. Following immune stimulation of *L. caerulea*: total globulins along with α-1 and γ globulin fraction concentrations increased, while the A–G ratio decreased; none of the plasma biochemical values changed significantly. In *L. infrafrenata*: plasma CK increased while glucose decreased post-immune stimulation; total protein, albumin, total globulins and α-1 globulin concentrations, and A–G ratio, all increased.

## Discussion

Our results provide the first direct evidence suggesting that *Bd* infection causes multiple *in vivo* systemic immune function defects in a susceptible amphibian host, which likely enables disease development. Antigenic stimulation of *L. caerulea* chronically infected with *Bd* resulted in lower splenic lymphocyte, WBC, serum protein and immunoglobulin responses compared with uninfected frogs. Although *L. infrafrenata* failed to maintain infection with *Bd* after experimental exposure, recovered frogs also had reduced WBC and serum globulin concentrations compared with unexposed frogs. Our work expands on recent studies showing *in vitro* lymphocyte proliferation was impaired by *Bd* cells and supernatants [31].

We successfully used diverse methods, many of which have not previously been used outside of applied veterinary research, to assess general innate and adaptive host immune competence in two tree frog species with and without *Bd* infection. All of the methods, with the exception of the PHA skin test, appeared to be reliable, and we present comprehensive baseline immune function data for the two frog species.

Following stimulation of uninfected *L. caerulea*, immune organ activation resulted in circulating WBC and protein responses, total (IgM and IgY) and IgY-specific serum antibody production, and splenic lymphocytic IgM-specific antibody production. These indicate active cellular and humoral responses consistent with those seen in other taxonomic groups [51], [52]. The change in RBC indices (reduced PCV, Hb, MCH and MCHC, concurrent with increased polychromasia) were not unexpected due to repeated blood sample collection within a short period of time, and indicate an adequate bone marrow regenerative response to relative anemia [48]. This range of data, combined with additional splenic cell measurements, provides comprehensive baseline immune function data for this species.

Compared with uninfected *L. caerulea*, chronic *Bd* infection in this species caused a significant reduction in total spleen cell concentration, splenic cell viability, splenic lymphocyte antibody production and liver-body weight ratio. The WBC, lymphocyte, monocyte and serum protein responses to immune stimulation were also reduced. All of these changes indicate an impaired ability of *Bd*-infected *L. caerulea* to respond adequately to antigenic stimulation. Inflammatory cells do not migrate to the sites of infection [26], hence the reductions in circulating white cell numbers in *Bd*-infected *L. caerulea* were most likely caused by defects in production and/or survival of these immune cells. The significance of the A–G ratio increase without a concurrent change in any of the five protein fractions remains unclear but may indicate a subtle serum protein response. Infected frogs had a lower Hb concentration decrease and polychromatophilic RBC response following immune stimulation. Although these two changes are likely to be a direct regenerative response to recent blood collection, the lower magnitude of these responses in the *Bd*-infected frogs may indicate reduced organ response to physiologic stimulation. The decreased mean thrombocyte count is most likely a response to recent blood collection and relative anemia, but may also represent depressed bone marrow function. Mean thrombocyte count also decreased in the uninfected frogs but this was not statistically significant.

Active cellular, humoral and splenic responses in unexposed *L. infrafrenata* were generally consistent with those in the uninfected *L. caerulea*, and in other taxonomic groups [52], [53]. Notable inter-species differences in our results include the neutrophilic versus lymphocytic WBC response and the hyperglycemia and elevated CK post-immune stimulation in *L. infrafrenata*. Neutrophil reference values in *L. infrafrenata* vary with season, and are significantly higher compared with *L. caerulea* [44]. Furthermore, the two species differ in temperament, with *L. caerulea* generally calm and tolerant of handling, and *L. infrafrenata* often exhibiting clinical signs of stress associated with handling and confinement. This is likely to account for the neutrophilic WBC response and the increased glucose and CK values in *L. infrafrenata*. The reduced RBC count concurrent with increased polychromatophilic response again was not unexpected due to repeated blood sample collection, and indicate an adequate bone marrow regenerative response.

All *Bd*-exposed *L. infrafrenata* failed to maintain infection despite identical experimental conditions to those for *L. caerulea*, and the five of nine frogs that tested positive early in the trial self-cured. The sampling protocols were completed despite the loss of infection, and results showed immunologic effects on some systemic responses resulting from *Bd* exposure. Post-immune stimulation WBC, neutrophil, polychromatophilic RBC, thrombocyte and α-1 globulin concentrations, and skin PHA response, were reduced in the *Bd*-exposed *L. infrafrenata* compared with uninfected frogs. However, immune stimulation caused similar total and IgY-specific serum antibody, splenic lymphocytic IgM-specific antibody, and splenic cellular, responses in both uninfected and *Bd*-exposed *L. infrafrenata*. Variations in RBC indices and plasma glucose were not unexpected as previously discussed.

In previous studies using the model species *Silurana tropicalis*, *Bd* infection appeared to cause down-regulation of some immune genes including those associated with Toll-like receptors, complement pathways, and B- and T-lymphocytes [27], [28]. Recently, soluble factors in *Bd* culture supernatant inhibited *in vitro* lymphocyte proliferation assays, but did not reduce macrophage activity [31]. Other studies found higher concentrations of urinary corticosterone metabolites and plasma corticosterone, in *Bd*-infected *L. wilcoxii* and *L. caerulea* respectively, indicating a physiological stress response [30], [62].

Stress hormones are known to alter normal WBC distribution, and peripheral leukocyte profiles consistent with a classical mammalian stress-related response include a relative neutrophilia, lymphopenia and eosinopenia [63]. Peripheral neutrophilic and eosinopenic responses have previously been reported in *Bd*-infected larval anurans (*Rana catesbeiana*), although lymphocyte abundance did not change [64]. Conflicting findings have been reported in post-metamorphic anurans: juvenile *L. chloris* showed relative peripheral neutropenic, eosinopenic and basophilic responses to *Bd* infection [20], while *L. caerulea* showed a lymphopenic response [62], [65]. The amphibian leukocyte response to stress varies according to species and many other intrinsic and extrinsic factors, including season and sex [44], [66], [67], making interpretation of leukocyte profiles alone difficult.

Previous laboratory and field studies show that *L. caerulea* is highly susceptible to *Bd* [11], [12], [68], but little data is available for *L. infrafrenata*. Our results indicate that *L. infrafrenata* is a naturally resistant host. Pre-exposure data collected from *L. infrafrenata* did not differ greatly from *L. caerulea*, hence our results do not suggest a clear mechanism for greater natural resistance in *L. infrafrenata*, which may be related to localized and/or innate immune defense mechanisms that we did not measure. The skin peptide profiles of *L. infrafrenata* and *L. caerulea* differ greatly [41] and this could partly explain inter-species differences in host resistance, although *L. infrafrenata* produces no known major antibiotic peptides compared with at least five that have been identified in *L. caerulea* [41].

The reduced WBC, neutrophil and globulin concentrations in exposed *L. infrafrenata* may indicate direct host immune system costs associated with infection clearance in frogs challenged post-metamorphosis and/or redirection of host resources away from systemic adaptive immune responses towards alternate immune mechanisms involved in combating infection. Larval common toads (*Bufo bufo*) experimentally exposed to low *Bd* doses usually died at or soon after metamorphosis without detectable infections, suggesting fitness costs attributable to exposure, control and clearance in the absence of extensive pathogen proliferation [69]. However, exposed *L. infrafrenata* were still able to produce serum antibody, and splenic cellular and antibody, responses of similar magnitude to uninfected frogs following immune stimulation. This suggests that adaptive immune responses may also play an important role in *Bd*-resistant host species.

Despite our findings that *Bd* causes multiple defects in systemic immune function in *L. caerulea*, an Australian survey found only 6.5% (13/199) of frogs with severe chytridiomycosis had concurrent acute secondary infectious diseases [68]. The low incidence of secondary infections may indicate that systemic immune suppression is not generalized and distinct components remain effective against opportunistic pathogen invasion, and/or that systemic immune response capability is not reduced below a critical point until late in the disease process. Total and IgY-specific serum antibody responses did not differ between uninfected and infected frogs of either species, suggesting that serum immunoglobulin response as a component of systemic adaptive immunity is not suppressed in chronic chytridiomycosis and that it may not be an important component of disease resistance. These findings further support the hypothesis that localized and/or innate immune defense mechanisms may be key factors.

Skin PHA stimulation was minimal and variable in contrast to other taxa and this test was not found to be a sensitive indicator of immune function in either of the two *Litoria* species in our study. There was a slightly greater response in *L. infrafrenata* compared with *L. caerulea*, but this was still minimal compared with other species and quite variable. The PHA skin response in adult *R. pipiens* was also found to be highly variable and less sensitive for detecting pesticide-related immune suppression compared with hemocyanin-specific antibody and whole blood chemiluminescence tests [39], although in the cane toad (*Rhinella marina*) the test was found to reliably quantify immune response in an assay validation study [40]. Splenic cell viability assessed by trypan blue exclusion resulted in lower mean viable cell counts in both of the *Litoria* species in our study compared with avian and mammalian species, but this test was still useful for comparing immune function between *Bd*-infected and uninfected frogs. Future adaptations of this method to improve viable cell harvest should include the use of an amphibian-specific isotonic diluent; mammalian isotonic solutions such as Hanks balanced salt solution are hypertonic to amphibian cells [70] and may cause cell damage. Although bisection of the spleen was necessary to perform multiple splenic tests, improved cell viability may also be achieved by processing the whole spleen for one single method.

A limitation associated with our experimental design is that the two experimental trials were not conducted simultaneously. The trial in uninfected frogs commenced as a pilot study to validate methodology prior to application in the trial with *Bd*-infected frogs, but insufficient specimens were available to include a negative control group in the infection trial. This limitation was largely compensated for by 1) standardizing timing of sample collection relative to initiation of immune tests, 2) ensuring experimental procedures were performed by the same person (SY) using identical analytic equipment, reagents and methodology, and 3) maintaining identical experimental laboratory and husbandry conditions. Additionally, a large concurrent experiment in our laboratory showed that immunity did not decrease in uninfected *L. caerulea* held in captivity and blood sampled three times over this period - with the exception of a mild but significant decrease in WBC and lymphocyte counts on day 30, no hematologic immune biomarkers varied significantly after 60 or 125 days [24]. Our baseline immune function data provide a valuable tool for progressing future immunologic and *Bd* pathogenicity studies in amphibians. General mechanisms of fungal immune suppression include inducing anti-inflammatory cytokines, decreasing pro-inflammatory cytokines and complement evasion [71]. Further work on understanding the mechanisms of *Bd* immune suppression is needed and may involve exposing frogs directly to identified pathogen-secreted factors and assessing immune function using the *in vivo* methods that we have shown here to be sensitive indicators.

Our results may explain the lack of an adaptive immune response to infection in some species [22], [24], [72], and suggest that if vaccine development or other immune modulation is attempted in future trials, it will be necessary to better understand the mechanisms of immune suppression in order to overcome it. While many of the immune tests we describe here will be valuable for use in future immunologic studies, the total and differential WBC and serum electrophoresis measurements, along with antigen-specific serum antibody assays, have particularly wide potential future applications as repeatable, ante-mortem methods for assessment of amphibian immune function.
